# Associations of Unmet Food and Housing Needs with Mental Health and Overall Perceived Health Among Women with HIV: Is There a Moderating Effect of Social Support?

**DOI:** 10.1089/whr.2024.0120

**Published:** 2025-04-21

**Authors:** Ekpereka Sandra Nawfal, Aaliyah Gray, Diana M. Sheehan, Sofia B. Fernandez, Tan Li, Robert Ladner, Mary Jo Trepka

**Affiliations:** ^1^ Department of Epidemiology, Florida International University, Miami, Florida, USA.; ^2^ Research Center in Minority Institutions, Florida International University, Miami, Florida, USA.; ^3^ School of Social Work, Robert Stempel College of Public Health and Social Work, Florida International University, Miami, Florida, USA.; ^4^ Department of Biostatistics, Academic Health Center 5, Florida International University, Miami, Florida, USA.; ^5^ Behavioral Science Research Corporation, Coral Gables, Florida, USA.

**Keywords:** HIV/AIDS, women with HIV, food insecurity, housing insecurity, mental health

## Abstract

**Background:**

Food and housing insecurity have been identified as modifiable risks for poor mental health and perceived self-rated health among people with HIV. This study examined the associations of food and housing insecurity with perceived overall health, depression and anxiety symptoms, and the potential moderating effect of social support.

**Methods:**

We conducted a cross-sectional study of 561 low-income women with HIV (WHIV) in the Miami-Dade County Ryan White Part A Program. Data were collected from June 2021 to March 2022. Food and housing insecurity were categorized into three groups: no food/housing insecurity, any food/housing insecurity, and concurrent food/housing insecurity. Multivariable logistic regression analyses were performed for each outcome variable.

**Results:**

Compared to no food/housing insecurity, significant depressive symptoms were associated with any food/housing insecurity (aOR: 2.99, 95% confidence interval [CI]: 1.81–4.91) and concurrent food/housing insecurity (aOR: 17.11, 95% CI: 7.83–37.38). Significant anxiety symptoms were associated with any food/housing insecurity (aOR: 3.50, 95% CI: 1.68–7.30) and concurrent food/housing insecurity (aOR: 15.97, 95% CI: 6.92–36.87). Although social support was significantly related to depressive and anxiety symptoms, it did not moderate the relationship between these unmet needs and any of the health outcomes.

**Conclusion:**

Our findings revealed significant associations between unmet food and housing needs, poor social support, and significant depressive and anxiety symptoms among WHIV. Continuous, multifaceted support is essential to mitigate the negative impact of unmet food and housing needs and ensure the physical and mental well-being of WHIV.

## Introduction

Food insecurity (limited ability to acquire adequate food due to a lack of money and other resources)^1^ and housing insecurity (encompassing issues such as affordability, safety, quality, instability, and homelessness)^2,3^ are two crucial social determinants of health that disproportionately affect people with HIV (PWH).^1,3,4^ Food insecurity and homelessness affect millions of people globally and are persistent major public health concerns in the United States (U.S.).^1,5–7^ In 2022, 12.8% (17.0 million) of U.S. households were food insecure, a statistically significant increase from 10.2% (13.5 million households) in 2021.^1^ As of January 2023, an estimated 653,104 people in the U.S. experienced homelessness (*i.e.,* lacking a fixed, regular, and adequate nighttime residence) on a single night, which was an increase of approximately 12% from 2022.^7^ A recent analysis of U.S. Medical Monitoring Project (MMP) data revealed that among people diagnosed with HIV in 2021, 17% reported unstable housing or homelessness, and 16% reported hunger or food insecurity during the 12 months prior to participants’ interviews, exceeding both national estimates for the general population and the 2025 National HIV/AIDS Strategy target of 11%.^3^ Gender differences were also observed, with 17.2% of women with HIV (WHIV) reporting unstable housing or homelessness compared to 16.6% of men with HIV (MHIV), and 16.2% of WHIV reporting hunger or food insecurity compared to 14.9% of MHIV.^3^

Food and housing insecurity are recognized modifiable risks of poor mental health such as depression and anxiety, and perceived self-rated health.^8–13^ Notably, in a large global study involving participants from 138 countries, living conditions—such as food insecurity and inadequate shelter or housing—were significantly associated with poorer physical health and low subjective well-being, with food insecurity showing the strongest level of association.^12^

The prevalence of depression and anxiety are disproportionately higher in PWH compared to the general population,^14–18^ particularly among WHIV.^14,19^ Studies using MMP data showed that 38.2% of WHIV experienced any depression (symptoms or diagnosis)^19^ and 23% of WHIV had generalized anxiety disorder (GAD) symptoms,^14^ compared with 24.0% depression diagnosis,^20^ and 19.0% GAD symptoms,^21^ among women in the general population. Women are also more likely than men to experience depression and GAD symptoms both in the general population and in PWH.^14,19–22^

The perception of general health, which refers to individuals’ subjective assessment of their overall health status,^23^ in PWH could be negatively influenced by their socioeconomic circumstances in addition to medical factors such as disease progression, treatment adherence, and comorbidities. A study found that PWH who reported experiencing food or housing insecurity had a lower prevalence of “good or better” self-rated health compared to PWH without food or housing insecurity.^24^

Further, in a longitudinal study with 602 PWH in Ontario, food insecurity was negatively associated with physical (effect size [ES] = −2.1, 95% confidence interval [CI] = −3.9, −0.3), and mental (*ES* = −3.5, CI = −6.1, −1.5) quality of life outcomes.^8^ A similar finding was reported in a qualitative study, where some participants recounted developing depression and anxiety due to food insecurity, or worsening of preexisting depression due to severe food insecurity and homelessness.^25^ Researchers have also identified a dose–response relationship between food insecurity and depressive and anxiety symptoms.^13,26–28^ This indicates that the impact of food insecurity on depressive and anxiety symptoms becomes stronger as the level of food insecurity increases from very low/low to severe.

Unmet subsistence needs often coexist among PWH, resulting in a more detrimental impact on their mental and physical health.^13,29,30^ This was demonstrated in a longitudinal study assessing the impact of competing risks on the health status among 129 unstably housed and homeless WHIV, which showed that unmet subsistence needs (*i.e.,* food, hygiene, and shelter needs) had the strongest negative effect on overall mental health.^29^ For WHIV, the intersectionality of gender-related issues, such as gender-based violence,^31,32^ discrimination,^33^ child care responsibility,^34^ low income,^35^ and HIV-related stigma,^36,37^ can compound the experiences of food insecurity and housing instability.^38,39^ Together, these experiences further diminish mental health and sense of overall well-being and can ultimately lead to suboptimal engagement in HIV care, antiretroviral therapy adherence, and HIV health outcomes.^25,38,40^

Having some source of support either from social networks (family, friends, or intimate partner) healthcare providers, or community organizations could help mitigate the impact of stresses related to unmet subsistence needs and promote adaptive coping strategies. Social support is theorized to buffer the effects of life stressors, including food and housing insecurity on physical and mental health status.^41–43^ Although, existing literature underscores the direct link of food and housing insecurity on mental health and general perceived health among PWH in the USA,^25,40,44^ the impact of these factors, especially the co-occurrence of both unmet subsistence needs, among WHIV vulnerable to multiple socioeconomic challenges, and the role of social support as a moderator or mediator remain underexplored. Understanding the relationships between unmet needs, mental and physical health, and the role of social support is crucial to providing additional insight into women-centered interventions that may help prevent comorbidities and address health inequalities.

This current study aims to examine the separate and concurrent associations of food and housing insecurity with general perceived health, depression, and anxiety among a cohort of WHIV receiving HIV care in the Miami-Dade County Ryan White Part A Program (RWP) and further explores the potential moderating effect of self-reported social support on the relationship between food and housing insecurity and these health outcomes.

## Materials and Methods

### Study design and data

This was a cross-sectional study that included a sample of 561 WHIV receiving services through the Miami-Dade County RWP. The RWP is a national service delivery network providing services as a payor of last resort to low-income persons with HIV. The survey was originally developed to analyze the role of patient-centered care on viral suppression, and details about its development are reported elsewhere.^45^ The survey was administered in English, Spanish, or Haitian Creole, based on participants’ preferences. Data were collected from June 2021 to March 2022 *via* telephone after verbal informed consent. The inclusion criteria were age ≥18 years, been in the RWP for at least 6 months, and being able to communicate in English, Spanish, or Haitian Creole. This study was approved by the Florida International University institutional review board.

### Measures

#### Predictor variables

The two predictor variables were food insecurity and housing insecurity. To measure food insecurity, we adapted a 1-item question from the U.S. Household Food Insecurity Survey.^46^ In the survey, respondents were asked: “The food that (I/we) bought just didn’t last, and (I/we) didn’t have money to get more. Was that often, sometimes, or never true for (you or your household) in the past 6 months?” We categorized participants responses into two: food insecure (“often” or “sometimes true”) and food secure (“never true”).

We measured housing insecurity using questions adapted from the Ryan White Client Satisfaction Survey (Personal communication; [Behavioral Science Research Corporation], February 13, 2020) and the Housing Status Assessment Tool utilized by the Department of Health and Human Services^47^: (i) What is your current housing situation? (own or rent your own house or apartment; live with someone else who owns or rents the house or apartment; renting a room in someone else’s house or apartment; couch surfing with friends or relatives, having a place to go at night but without a permanent address; homeless, on street, in a shelter or in temporary housing; in a treatment facility or hospital). (ii) Over the past 12 months, have you stayed in a shelter, transitional housing, or places like a hotel or motel paid for by a social service or charitable organization, or stayed in a place not meant for human habitation, such as the streets, parks, or abandoned buildings? (yes/no). (iii) Over the past 12 months, have you been at risk of losing housing or lost housing because of difficulty paying rent or eviction? (yes/no). (iv) Over the past 12 months, have you been at risk of losing housing or lost housing because of health/safety concerns, being asked to leave, host family/friend risk, or conflict? (yes/no). Participants who reported currently couch surfing, homeless on the street, in shelter, temporary housing, treatment facility, or hospital or answered “yes” to any of the questions 2–4 were classified as experiencing housing insecurity, and all others were classified as not experiencing housing insecurity.

We created three mutually exclusive groups to analyze the individual and combined associations of food and housing insecurity with the outcome variables: (i) participants who did not experience food and housing insecurity (no food/housing insecurity); (ii) those who experienced either food insecurity or housing insecurity (any food/housing insecurity); and (iii) those who experienced both food and housing insecurity (concurrent food/housing insecurity).

### Moderator variable

#### Social support

Social support was measured using a single instrument from the AIDS Clinical Trials Group Questionnaire.^48^ Participants were asked, *“*In general, how happy are you with the overall social support (help) that you get from your family and friends?”. The response options were “not at all happy,” “somewhat happy,” “moderately happy,” “very happy,” and “extremely happy.” We dichotomized the response into: “not at all/somewhat happy” and “moderately/very/extremely happy.”

### Outcome variables

#### Depressive symptoms

A short version of the Center for Epidemiologic Studies Depression scale (CES-D)^49^ was used to measure depression; the CES-D-10 consists of 10 self-reported items, which assess the frequency of depressive symptoms over the past week. The CES-D-10 is a screening instrument that has been demonstrated to have good predictive accuracy when compared to the 20-item CES-D version and has been used with PWH.^50,51^ Each item includes four response categories ranging from ‘‘rarely or none of the time (less than 1 day)’’ to ‘‘most or all of the time (5–7 days).’’ Item scores were totaled to generate a possible summary score ranging from 0 to 30. We dichotomized the score using a recommended cutoff score of ≥10,^49^ as having significant depressive symptoms. The Cronbach’s alpha for the 10 depression items in this study was 0.84, indicating high internal consistency.

#### Anxiety symptoms

Anxiety symptoms were assessed using the GAD-7 scale,^52^ a valid and efficient tool for screening for GAD. The GAD-7 scale consists of 7 items with a 4-point Likert-format response that reflects major symptoms related to being nervous and restless in the past 2 weeks. Each of the items was scored from 0 to 3, and the total score ranges from 0 to 21. For this study, the total score was categorized into two groups using the cutoff point of 10,^52,53^ (<10 = minimum-to-mild anxiety symptoms and ≥10 = moderate-to-severe anxiety symptoms [hereinafter referred to as significant anxiety symptoms]). The Cronbach’s alpha for the GAD-7 scale was 0.91, indicating high internal consistency.

#### General perceived health

A single self-rated item from the RAND Health Survey,^54^ was used to measure participants’ perception of their health status that consisted of asking respondents to rate their health as ‘‘excellent,” “very good,” “good,” “fair,” or “poor.’’ For our analysis, the response was classified into two categories: “fair/poor” versus “good/very good/excellent.”

### Covariates

Demographic variables that were included in the analysis due to their potentially confounding effects were age, race/ethnicity, and education. Participant age was categorized into three groups: young (18–34 years), middle-aged (35–49 years), and older adults (≥50 years); race/ethnicity into four groups: non-Hispanic Black (excluding Haitians), Hispanic (of any race), Haitian (of any race), or other race/ethnicity (non-Hispanic White, Asian, American Indian, and Alaska Native); and educational level into three groups: less than high school, high school graduate, and trade school/any college. Other variables that were included were employment status (employed vs. unemployed); number of minors (children younger than 18 years) dependent on participant (none vs. one or more); household income, which was classified into <100% U.S. Federal Poverty Level (FPL), 100%–199% FPL, and ≥200% FPL; and number of years since HIV diagnosis categorized into three groups: <10 years, 10–19 years, and ≥20 years.

### Statistical analysis

All analyses were conducted using Statistical Analysis System (SAS) software, version 9.4 (SAS Institute, Cary, NC). Frequencies and proportions were utilized to describe the distribution of the variables of interest (Table 1). Bivariate analyses were performed using the chi-squared (χ^2^) test to compare the differences in variables across the three outcome variables: general perceived health, depressive symptoms, and anxiety symptoms with *p* < 0.05 (two-tailed) considered statistically significant (Table 1).

**Table 1.** Characteristics of Study Participants and Bivariate Association with Outcome Variables (General Perceived Health, Significant Depressive Symptoms, and Significant Anxiety Symptoms) (*n* = 561)

		General perceived health			Significant depressive symptoms			Significant anxiety symptoms		
Characteristics	Total, *n* (column %)	Good/very good/excellent, *n* (row %)	Fair/poor, *n* (row %)	*p* ^a^	<10, *n* (row %)	≥10, *n* (row %)	*p* ^a^	<10, *n* (row %)	≥10, *n* (row %)	*p* ^a^
Total		495 (88.2)	66 (11.8)		398 (74.4)	137 (25.6)		473 (88.1)	64 (11.9)	
Age group				0.9147			**0.0421**			**0.0059**
18–34	36 (6.4)	31 (86.1)	5 (13.9)		22 (61.1)	14 (38.9)		26 (72.2)	10 (27.8)	
35–49	169 (30.1)	149 (88.2)	20 (11.8)		113 (70.6)	47 (29.4)		142 (87.1)	21 (12.9)	
≥50	356 (63.5)	315 (88.5)	41 (11.5)		263 (77.6)	76 (22.4)		305 (90.2)	33 (9.7)	
Race/ethnicity^a,b^				**0.0300** ^a^			**<.0001**			**0.0195** ^a^
Black	198 (35.3)	164 (82.8)	34 (17.2)		135 (68.5)	62 (31.5)		168 (85.3)	29 (14.7)	
Hispanic	186 (33.2)	167 (89.8)	19 (10.2)		129 (70.5)	54 (29.5)		159 (85.5)	27 (14.5)	
Haitian	158 (28.2)	146 (92.4)	12 (7.6)		121 (88.9)	15 (11.0)		129 (95.6)	6 (4.4)	
Non-Hispanic White	19 (3.4)	18 (94.7)	1 (5.3)		13 (68.4)	6 (31.6)		17 (89.5)	2 (10.5)	
Household income (percent of Federal Poverty Level)				0.2877			**0.0392**			0.2448
<100%	245 (43.8)	212 (86.5)	33 (13.5)		162 (69.2)	72 (30.7)		205 (87.2)	30 (12.8)	
100%–199%	199 (35.5)	175 (87.9)	24 (12.1)		146 (76.8)	44 (23.1)		166 (86.5)	26 (13.5)	
≥200%	116 (20.7)	107 (92.2)	9 (7.8)		90 (81.1)	21 (18.9)		101 (92.6)	8 (7.3)	
Educational level				0.2639			**0.0463**			0.5295
Less than 12th grade	164 (29.3)	141 (85.9)	23 (14.0)		113 (75.3)	37 (24.7)		138 (90.2)	15 (9.8)	
High school graduate	212 (37.9)	185 (87.3)	27 (12.7)		163 (79.1)	43 (20.9)		179 (88.2)	24 (11.8)	
College or trade school	184 (32.9)	168 (91.3)	16 (8.7)		122 (69.2)	57 (31.8)		156 (86.2)	25 (13.8)	
Employment status				0.1945			0.5886			0.9675
Unemployed	264 (47.1)	228 (86.4)	36 (13.6)		184 (73.3)	67 (26.7)		223 (88.1)	30 (11.9)	
Employed	297 (52.9)	267 (89.9)	30 (10.1)		214 (75.4)	70 (24.7)		250 (88.0)	34 (11.9)	
Number of children				0.2148			0.1490			0.0740
None	403 (71.9)	352 (87.3)	51 (12.6)		292 (76.2)	91 (23.8)		344 (89.8)	39 (10.2)	
One or more	157 (28.0)	143 (91.1)	14 (8.9)		106 (70.2)	45 (29.8)		129 (84.3)	24 (15.7)	
Years since HIV diagnosis				**0.0490**			0.1618			0.9066
<10	214 (38.2)	183 (85.5)	31 (14.5)		141 (69.8)	61 (30.2)		182 (87.9)	25 (12.1)	
10–19	213 (37.9)	186 (87.3)	27 (12.7)		155 (76.7)	47 (23.3)		176 (87.6)	25 (12.4)	
≥20	134 (23.9)	126 (94.0)	8 (5.9)		102 (77.9)	29 (22.1)		115 (89.2)	14 (10.9)	
Food and housing insecurity 0.0302							**<0.0001**			**<0.0001**
No food/housing insecurity	330 (59.9)	299 (90.6)	31 (9.4)		271 (87.4)	39 (12.6)		301 (96.2)	12 (3.8)	
Any food/housing insecurity	170 (30.9)	147 (86.5)	23 (13.5)		108 (65.5)	57 (34.6)		137 (84.05)	26 (15.9)	
Concurrent food/housing insecurity	51 (9.3)	40 (78.4)	11 (21.6)		12 (23.5)	39 (76.5)		27 (52.9)	24 (47.1)	
Happy with overall social support				**0.0048**			**<0.0001**			**<0.0001**
Not at all/somewhat happy	53 (10.3)	40 (75.5)	13 (24.5)		22 (42.3)	30 (57.7)		35 (66.0)	18 (33.9)	
Moderately/very/extremely happy	462 (89.7)	411 (88.9)	51 (11.0)		342 (76.9)	103 (23.2)		403 (89.7)	46 (10.2)	

*Note:* Missing responses: household income (*n* = 1), education level (*n* = 1), number of children (*n* = 1), food and housing insecurity (*n* = 10), overall social support (*n* = 46).

^a^ *p*-values for all variables were obtained from chi-squared tests, except for the *p*-values for the association between race/ethnicity and general perceived health and anxiety, which was obtained from Fisher’s exact tests. Significant *p*-values (*p* ≤ 0.05) are bolded.

^b^ Non-Hispanic Black group excludes Haitians.

Univariate and multivariable logistic regression analyses were conducted to assess the associations between the main predictors (food and housing insecurity) and the outcome variables, and the potential moderating effect of social support. Covariates selection was based on previous studies that have linked these variables to the predictors or outcomes.^27,55^ Variables with *p* value <0.2 in the bivariate analyses for each outcome variable were included in the respective logistic regression model. To address data missingness, we conducted multiple imputations using the fully conditional specification method. We used the imputed dataset for the logistic regression analyses and then utilized the pooled estimates from the 20 imputed datasets. Furthermore, to avoid problems with collinearity, variance inflation factor (VIF) was used to detect multicollinearity, with a VIF cutoff of 10 indicating signs of serious multicollinearity.^56^

We fit three multiple logistic regression models for each outcome variable. The main predictors (food and housing insecurity) were included in the first model. In the second model, we included the main predictors along with the moderator (social support). Subsequently, the product terms for the interactions between the main predictors and moderator were entered in the third model to assess the extent to which the estimated strength of the associations between any food/housing insecurity, concurrent food/housing insecurity, and the outcome variables differed according to the overall social support. Covariates were included in the models to control for potential confounding effects.

## Results

### Study population

The mean age of participants was 52.2 years (SD = 10.6; range: 22–90 years) with over half of the participants being 50 years and older (63.5%) (Table 1). An almost equal proportion of the participants identified as non-Hispanic non-Haitian Black (35.3%) and Hispanic (33.2%). Approximately 29% had less than a high school education, 53% were employed, and 44% had an annual income that was below 100% of the FPL. The majority had no minor children (71.9%), reported no food/housing insecurity (59.9%), and were moderately/very/extremely happy with the overall social support received from family and friends (89.7%). The mean years since receiving their HIV diagnosis was 13.8 years (SD = 8.2). There was no evidence of multicollinearity between any of the variables. All VIF values were <10.

### Associations between unmet needs, social support with general perceived health, depression, and anxiety

#### General perceived health

Among the 561 participants, 11.8% perceived their general health as “fair/poor” (Table 1). In the bivariate analyses (Table 1), race/ethnicity, number of years since HIV diagnosis, food and housing insecurity, and overall social support were significantly associated with general perceived health (*p* < 0.05). In the unadjusted analyses (Table 2), only concurrent food/housing insecurity and social support were significantly associated with general perceived health. Those who reported experiencing concurrent food/housing insecurity (OR: 2.64, 95% CI: 1.23–5.65) compared to no food/housing insecurity, and those “not at all or somewhat happy” versus “moderately/very/extremely happy” with their overall social support (OR: 2.69, 95% CI: 1.35–5.33) were more likely to rate their general perceived health as “fair/poor.” In adjusted analyses, controlling for race/ethnicity, employment status, and years since HIV diagnosis, concurrent food/housing insecurity was no longer significant (Table 2, model 1). Concurrent food/housing insecurity remained nonsignificant when social support was included in the regression model (Table 2, model 2). The interaction terms between the unmet subsistence needs (food and housing insecurity) and social support were not significant (Supplementary Table S1), indicating a lack of the moderating effect of social support on the association between the unmet subsistence needs and general perceived health.

**Table 2.** Results of Logistic Regression Analysis for Self-Rated General Perceived Health (Fair/Poor), Reference Group: Good/Very Good/Excellent

		Adjusted models	
	Unadjusted model	Model 1	Model 2
Variable	OR (95% CI)	aOR (95% CI)	aOR (95% CI)
Food and housing insecurity			
Any food/housing insecurity versus no food/housing insecurity	1.50 (0.85–2.67)	1.32 (0.73–2.39)	1.23 (0.67–2.23)
Concurrent food/housing insecurity versus no food/housing insecurity	**2.64 (1.23–5.65)**	2.06 (0.93–4.55)	1.64 (0.71–3.79)
Social support			
Not at all/somewhat happy versus moderately/very/extremely happy	**2.69 (1.35–5.33)**	—	2.09 (0.99–4.41)

Covariates included in the adjusted models are race/ethnicity, employment status, and years since HIV diagnosis.

CI = confidence interval.

*Note*: Significant odd ratios are bolded.

#### Mental health

Approximately 26% of 535 WHIV who responded to depression questions had significant depressive symptoms, and among the 537 WHIV who responded to anxiety questions, 12% reported having significant anxiety symptoms (Table 1). Results from the bivariate analyses showed significant associations between having significant depressive symptoms and age, race/ethnicity, income level, educational level, food and housing insecurity, and social support. Significant anxiety symptoms were significantly associated with age, race/ethnicity, food and housing insecurity, and social support.

Table 3 summarizes the unadjusted and adjusted logistic regression results for significant depressive symptoms. The unadjusted models revealed that having significant depressive symptoms was associated with any food/housing insecurity (OR: 3.74, 95% CI: 2.33–6.01), concurrent food/housing insecurity (OR: 22.23, 95% CI: 10.71–46.17), and dissatisfaction with social support (OR: 4.53, 95% CI: 2.51–8.16). In the adjusted models, the same factors remained significant (Table 3). The interaction terms between food/housing insecurity and social support for significant depressive symptoms were not statistically significant (Supplementary Table S2), indicating that there was no moderating effect of social support.

**Table 3.** Results of Logistic Regression Analysis for Significant Depressive Symptoms (**≥**10 on the Center for Epidemiological Study Depression-10 Scale), Reference Group: **<**10

		Adjusted models	
	Unadjusted model	Model 1	Model 2
Variable	OR (95% CI)	aOR (95% CI)	aOR (95% CI)
Food and housing insecurity			
Any food/house insecurity versus no food/housing insecurity	**3.74 (2.33–6.01)**	**3.25 (1.98–5.33)**	**2.99 (1.81–4.91)**
Concurrent food/housing insecurity versus no food/housing insecurity	**22.23 (10.71–46.17)**	**20.49 (9.48–44.29)**	**17.11 (7.83–37.38)**
Social support			
Not at all/somewhat happy versus moderately/very/extremely happy	**4.53 (2.51–8.16)**	—	**2.74 (1.37–5.50)**

Covariates included in the adjusted models are age, race/ethnicity, household income, educational level, number of children, and years since HIV diagnosis.

CI = confidence interval.

*Note*: Significant odd ratios are bolded.

Compared to having no food/housing insecurity, any food/housing insecurity (OR: 4.56, 95% CI: 2.25–9.27) and concurrent food/house insecurity (OR: 21.02, 95% CI: 9.55–46.27) were associated with significant anxiety symptoms in the unadjusted model. Further, dissatisfaction with social support was associated with significant anxiety symptoms (OR: 4.51, 95% CI: 2.36–8.62) (Table 4). These variables remained significant in the adjusted models, with covariates and food and housing insecurity variables included in model 1, and the social support variable additionally included in model 2 (Table 4). The interaction terms between food/housing insecurity and social support for significant anxiety symptoms were not statistically significant (Supplementary Table S3), suggesting that social support was not a significant moderator.

**Table 4.** Results of Logistic Regression Analysis for Significant Anxiety Symptoms (**≥**10 on Generalized Anxiety Disorder-7 Scale), Reference Group: **<**10

		Adjusted models	
	Unadjusted model	Model 1	Model 2
Variable	OR (95% CI)	aOR (95% CI)	aOR (95% CI)
Food and house insecurity			
Any food/house insecurity only versus no food/housing insecurity	**4.56 (2.25–9.27)**	**3.89 (1.88–8.04)**	**3.50 (1.68–7.30)**
Concurrent food/housing insecurity versus no food/housing insecurity	**21.02 (9.55–46.27)**	**19.78 (8.73–44.82)**	**15.97 (6.92–36.87)**
Social support			
Not at all/somewhat happy versus moderately/very/extremely happy	**4.51 (2.36–8.62)**	—	**2.51 (1.19–5.33)**

Covariates included in the adjusted models are age, race/ethnicity, and number of children.

CI = confidence interval.

*Note*: Significant odd ratios are bolded.

## Discussion

Intersections of gender-related sociodemographic vulnerabilities,^33–37^ gender-based violence,^31,32^ and familial responsibilities^34^ leave WHIV particularly vulnerable to poorer physical health, unmet subsistence needs, and heightened mental health concerns.^25,38–40^ Among our sample of WHIV, fair/poor self-rated health, and significant anxiety symptoms were reported among 12% of participants. Moreover, a quarter reported significant depressive symptoms. These findings are somewhat consistent with previous reports from United States MMP data, demonstrating favorable self-rated health (60.7%)^3^ and a depression prevalence of 38.2%^19^ among WHIV. However, three times as many women in our sample reported significant anxiety symptoms in the past 2 weeks compared to a past-year prevalence of 4% among a general sample of women (*i.e.,* HIV serostatus not reported) from a 2011 study.^22^ Our data reflect that mental health is poorer among WHIV than the general population,^20–22^ while general perceived health is likely higher.^57,58^ Compared to CDC data on unmet needs among WHIV,^3^ a higher percentage of our sample—approximately 40% overall—reported at least one unmet food or housing need, and of those, about 9% experienced concurrent food and housing insecurity.

The first aim of this study was to examine whether unmet needs were associated with self-reported general perceived health, depressive symptoms, and anxiety symptoms. Unmet food and housing needs have been consistently associated with poorer physical health outcomes and greater mental health difficulties.^8–13,24–30^ Consistent with these previous studies, WHIV in our sample experiencing unmet food or housing needs, particularly those facing both food and housing insecurity, were more vulnerable to higher depressive and anxiety symptoms. By contrast, we found that only concurrent food and housing insecurity was associated with poorer perceived health, although this association did not remain significant when controlling for the sociodemographic covariates and social support. Lack of association with perceived general health is likely explained by high levels of treatment adherence and viral suppression among our sample,^45^ contributing to an overall stronger perceived physical well-being.^25,38–40^ Those providing coordinated care for WHIV should screen for both mental health difficulties and unmet needs. Timely referrals for appropriate support services and resources must be provided as an integral part of case management for those reporting unmet subsistence needs. As food and housing costs continue to rise, the proportion of WHIV, particularly older WHIV, reporting unmet subsistence needs will likely continue to grow as a predominant issue facing WHIV.

Food banks and other food access resources available through local, state, and federal programs are beneficial for WHIV experiencing food insecurity. Between fiscal years 2021 and 2022, the expenditures for the food bank services provided by the Miami-Dade County RWP nearly doubled with an increase from $1.3 million to $2.5 million in funds spent.^59^ Housing support services, however, are more costly and fewer federal, state, and community-based resources may be available. There is a great need for affordable housing in major metropolitan areas such as Miami-Dade County, where the cost of living, housing instability, and risk of becoming unhoused are increasing.^60^ Medical case managers should be aware of services to support housing needs such as public housing and housing choice vouchers, subsidized rent and financial assistance programs, the Housing Opportunities for Persons With AIDS Program, and community-based resources available to WHIV.^61^ Until the issue of lack of affordable housing is adequately addressed, it is important that providers working with WHIV should be aware of waiting lists and other barriers to receiving these services as well. Beyond that, funding for community-based resources that provide other types of support, such as career and job placement services that can improve employment opportunities among WHIV, is needed.

We also explored social support as a moderator in the associations between unmet subsistence needs and general perceived health, depression, and anxiety. Social networks, including family, friends, HIV care providers, and community organizations, are thought to buffer the negative effects of life stressors on physical and mental health.^41–43^ Although we did not find a significant interaction between social support and unmet subsistence needs for any of the outcomes, we did find that being unhappy with overall social support was associated with increased odds of significant depressive and anxiety symptoms. Our findings indicate that in addition to subsistence support, WHIV presenting with mental health difficulties and/or social isolation might also benefit from opportunities for no- or low-cost support groups, psycho-educational interventions, peer programs, and community-based resources that aim to improve feelings of social support, belongingness, and sense of community which has been associated with promoting psychosocial well-being and mental health.^62,63^

There are several limitations to report. It is likely that selection bias exists in our sample because women more engaged in care were more likely to elect to participate in our study. As such, these findings may not be generalizable to WHIV not currently receiving HIV care or those who are not clients of the Miami-Dade County RWP. In addition, it should be noted that the majority of participants reported no unmet subsistence needs, and those experiencing housing insecurity, in particular, were underrepresented in our sample. Moreover, it is important to note that a single-item question was used to measure overall social support and it did not allow us to characterize the type of support (*i.e.,* emotional, instrumental, or informational social support) received.^41,43^ Further, causality between factors cannot be determined due to the cross-sectional nature of these data, and mental health status may affect perceptions of both social support and food and housing insecurity.^64,65^

## Conclusion

Targets for the 2025 National HIV/AIDS Strategy include reducing food and housing insecurity to 11%. However, available population-level data indicate that unmet subsistence needs among PWH exceed national estimates for the general population and that food and housing issues will continue to increase, with aging populations on fixed incomes most vulnerable to adverse outcomes. Our findings contribute to this literature by demonstrating significant associations between unmet food and housing needs, poor social support, and significant depressive and anxiety symptoms. WHIV experiencing unmet needs and social difficulties are in need of appropriate services that can improve and promote food security, housing stability, and better mental health outcomes. Federal, state programs, and local programs as well as community-based organizations are central to resolving these issues and require substantial funding to do so. Case managers and other HIV care providers must be able to support WHIV in navigating and securing these much-needed resources and services to ensure their physical and mental health.

## Abbreviations Used

CES-D: Center for Epidemiologic Studies Depression
FPL: Federal Poverty Level
GAD: Generalized Anxiety Disorder
HIV/AIDS: Human Immunodeficiency Virus/Acquired Immunodeficiency Syndrome
MHIV: Men with HIV
MMP: Medical Monitoring Project
PWH: People with HIV
RWP: Ryan White Program
VIF: Variance Inflation Factor
WHIV: Women with HIV
