# Supplementary material for: Associations of Unmet Food and Housing Needs with Mental Health and Overall Perceived Health Among Women with HIV: Is There a Moderating Effect of Social Support?
Source: Womens Health Rep (New Rochelle). 2025 Apr 21;6(1):453–63. doi: 10.1089/whr.2024.0120 (PMC12165826; doi:10.1089/whr.2024.0120)
Supplement: Supplementary Table S1 [file whr.2024.0120_supplementary_table_s1.docx]

| **Table SA1: Moderation of the association between food and housing insecurity and self-rated general perceived health (fair/poor) by social support** | |
| --- | --- |
|  | **Adjusted model** |
|  | **Model 3** |
| **Variable** | **aOR (95% CI)** |
| **Food and housing insecurity** |  |
| Any food/housing insecurity vs No food/housing insecurity | 1.21 (0.63 – 2.33) |
| Concurrent food/housing insecurity vs No food/housing insecurity | 2.12 (0.82 – 5.50) |
| **Social support** |  |
| Not at all/somewhat happy vs Moderately/very/extremely happy | 2.68 (0.73 – 9.84) |
| **Interaction terms** |  |
| Any food/house insecurity x social support (**ref:** no food/housing insecurity x moderately/very/extremely happy) | 0.93 (0.16 – 5.37) |
| Concurrent food/housing insecurity x social support (**ref:** no food/housing insecurity x moderately/very/extremely happy) | 0.43 (0.06 – 2.96) |

Covariates included in the adjusted models are race/ethnicity, employment status, and years since HIV diagnosis.  *Note:* ref. = reference group
