# Supplementary material for: Associations of Unmet Food and Housing Needs with Mental Health and Overall Perceived Health Among Women with HIV: Is There a Moderating Effect of Social Support?
Source: Womens Health Rep (New Rochelle). 2025 Apr 21;6(1):453–63. doi: 10.1089/whr.2024.0120 (PMC12165826; doi:10.1089/whr.2024.0120)
Supplement: Supplementary Table S2 [file whr.2024.0120_supplementary_table_s2.docx]

| **Table SA2: Moderation of the association between food and housing insecurity and significant depressive symptoms (> 10 on the Center for Epidemiological Study Depression–10 scale) by social support** | |
| --- | --- |
|  | **Adjusted model** |
|  | **Model 3** |
| **Variable** | **aOR (95% CI)** |
| **Food and housing insecurity** |  |
| Any food/house insecurity vs No food/housing insecurity | 2.56 (1.51 – 4.33) |
| Concurrent food/housing insecurity vs No food/housing insecurity | 26.66 (10.39 – 68.34) |
| **Social support** |  |
| Not at all/somewhat happy vs Moderately/very/extremely happy | 2.04 (0.56 – 7.44) |
| **Interaction terms** |  |
| Any food/house insecurity x social support (**ref:** no food/housing insecurity x moderately/very/extremely happy) | 3.26 (0.62 – 17.18) |
| Concurrent food/housing insecurity x social support (**ref:** no food/housing insecurity x moderately/very/extremely happy) | 0.26 (0.04 –1.77) |

Covariates included in the adjusted models are age, race/ethnicity, household income, educational level, number of children, and years since HIV diagnosis. *Note:* ref. = reference group
