# Supplementary material for: Associations of Unmet Food and Housing Needs with Mental Health and Overall Perceived Health Among Women with HIV: Is There a Moderating Effect of Social Support?
Source: Womens Health Rep (New Rochelle). 2025 Apr 21;6(1):453–63. doi: 10.1089/whr.2024.0120 (PMC12165826; doi:10.1089/whr.2024.0120)
Supplement: Supplementary Table S3 [file whr.2024.0120_supplementary_table_s3.docx]

| **Table SA3: Moderation of the association between food and housing insecurity and significant anxiety symptoms (> 10 on Generalized Anxiety Disorder-7 scale) by social support** | |
| --- | --- |
|  | **Adjusted models** |
|  | **Model 3** |
| **Variable** | **aOR (95% CI)** |
| **Food and house insecurity** |  |
| Any food/house insecurity only vs No food/housing insecurity | 2.88 (1.28 – 6.49) |
| Concurrent food/housing insecurity vs No food/housing insecurity | 25.24 (10.06 – 63.35) |
| **Social support** |  |
| Not at all/somewhat happy vs Moderately/very/extremely happy | 2.88 (0.42 – 19.77) |
| **Interaction terms** |  |
| Any food/house insecurity x social support (**ref:** no food/housing insecurity x moderately/very/extremely happy) | 2.12 (0.23 – 19.39) |
| Concurrent food/housing insecurity x social support (**ref:** no food/housing insecurity x moderately/very/extremely happy) | 0.21 (0.02 – 2.07) |

Covariates included in the adjusted models are age, race/ethnicity, and number of children.

*Note:* ref. = reference group
